# Cardiac Structural and Functional Evaluation Using a Heart Motion Correction Algorithm for Coronary Computed Tomography Angiography in Patients With High Heart Rates

**DOI:** 10.31083/RCM48026

**Published:** 2026-05-18

**Authors:** Xiaorong Chen, Yanping Dong, Aiyun Sun, Hao Xu, Xinyang Ge, Ronghua Wu, Shanshan Ying, Xiu Zhang, Jing Yuan, Jiangfeng Pan

**Affiliations:** ^1^Department of Medical Imaging, Affiliated Jinhua Hospital, Zhejiang University School of Medicine, 321000 Jinhua, Zhejiang, China; ^2^CT Imaging Research Center, GE HealthCare China, 200010 Shanghai, China; ^3^College of Mathematical Medicine, Zhejiang Normal University, 321004 Jinhua, Zhejiang, China

**Keywords:** coronary computed tomography angiography, motion correction, heart segmentation, strain

## Abstract

**Background:**

The heart motion correction algorithm used in current multi-slice computed tomography (CT) is sufficient for coronary artery imaging in patients with high heart rates. However, the effect of this algorithm on the image quality in whole-cardiac-cycle reconstructions remains unclear. Therefore, this study aimed to investigate image quality, segmentation performance, and cardiac structure and function assessment using a heart motion correction algorithm for coronary CT angiography in patients with rapid heart rates.

**Methods:**

This study retrospectively collected data from 58 consecutive patients with high heart rates (≥80 beats/min), of whom 36 also underwent cardiac magnetic resonance (CMR) imaging. CT images were reconstructed from 0% to 100% in 5% increments using the standard reconstruction (STD) and second-generation snapshot freeze (SSF2) protocols, and then processed by an automatic heart segmentation algorithm. Image quality, segmentation performance, cardiac volumes, and functional parameters were compared between protocols.

**Results:**

Compared with the STD protocol, the SSF2 protocol yielded a higher image quality score (3.91 ± 0.29 vs. 3.84 ± 0.37; *p* < 0.01), a steeper edge rise slope (41.71 ± 19.03 vs. 25.59 ± 13.16; *p* < 0.01), and lower entropy (4.12 ± 0.48 vs. 4.40 ± 0.28; *p* < 0.01). For left ventricular end-diastolic volume, the intraclass correlation coefficient (ICC) between automatic segmentation and manual contouring for the SSF2 protocol was 0.96, and the coefficient of variation was 7.84%. In contrast, the coefficients of variation for left ventricular end-systolic volume were poor (48.24% for STD and 48.18% for SSF2). Differences in global circumferential strain (–13.30 ± 3.42 vs. –15.01 ± 4.44; *p* < 0.01) and global longitudinal strain (–11.80 ± 4.83 vs. –13.01 ± 4.36; *p* < 0.01) between SSF2 and CMR were statistically significant, although correlations (ICC = 0.90 and 0.85, respectively) were good.

**Conclusions:**

SSF2 significantly improves image quality, structure, and function, and enables strain assessment in whole-cardiac-cycle reconstructions in patients with high heart rates. SSF2 also demonstrates superior performance over the STD protocol for evaluating myocardial strain.

## 1. Introduction

Coronary computed tomography angiography (CCTA) is widely used in the diagnosis and evaluation of cardiovascular diseases, including coronary heart disease, congenital heart disease, valvular abnormalities, and atrial fibrillation [1, 2, 3, 4, 5]. Elevated heart rate increases the incidence of motion artifacts and decreases image quality. Recent studies have revealed that the heart motion correction algorithm yields satisfactory images in patients with high heart rates [6]. The second-generation snapshot freeze (SSF2) algorithm—a novel deep learning-based heart motion correction method integrated into CCTA reconstruction—has enhanced coronary artery imaging in patients with increased heart rate [7]. Furthermore, SSF2 effectively stabilizes the left atrium, left atrial appendage, heart valve, and other structures, thereby improving diagnostic capabilities [1, 5].

Heart segmentation on CCTA is widely used to analyze cardiac structure and function [8, 9]. Manual segmentation remains feasible and strongly correlates with cardiac magnetic resonance (CMR) and echocardiography in evaluating atrial and ventricular volumes and ejection fraction in patients with moderate or low heart rate [8, 9, 10]. Achieving high-quality CCTA images in patients with high heart rates remains challenging. SSF2 has improved image quality during systole more effectively than during diastole in single-phase reconstruction of the valve and left atrium in patients with high heart rates [5]. However, its applicability to whole-cardiac-cycle reconstruction for functional analysis remains uncertain.

The whole-cardiac-cycle reconstruction of CCTA enables cardiac function and myocardial strain evaluation in patients with conditions such as coronary heart disease, valvular disease, hypertrophic cardiomyopathy, atrial fibrillation, and congenital heart disease [2, 4, 11, 12, 13]. However, results from different studies have indicated considerable variability. Recent studies revealed a strong correlation between CCTA, echocardiography, and CMR in the analysis of cardiac function, myocardial strain [4, 10, 14], and prognostic value [1, 15]. However, some studies have reported discrepancies in functional and strain parameters across modalities [10, 16, 17, 18]. There is a good reproducibility of myocardial strain measurements at moderate or slow heart rates (69 ± 12 beats/min) [19]; however, the impact of high heart rates on myocardial strain remains underinvestigated. Unlike single-phase reconstruction for coronary artery assessment, whole-cardiac-cycle reconstruction encompasses the full cardiac cycle. When the heart rate is ≥80 beats/min, the rapid contraction and relaxation of the heart—particularly during the rapid ejection and filling phase—pose significant challenges for cardiac motion freezing. Currently, no study has assessed cardiac function using heart motion correction algorithms or SSF2 in patients with high heart rates, thereby leaving an important gap in the literature.

This study aimed to investigate the application of SSF2 in enhancing CCTA image quality and heart segmentation, as well as the assessment of cardiac structure and function and myocardial strain in patients with high heart rates.

## 2. Materials and Methods

### 2.1 Patient Population

This retrospective study consecutively included 22,286 patients who underwent CCTA examination between September 2023 and April 2025. The inclusion criteria were a heart rate of ≥80 beats/min with sinus rhythm and the use of a retrospective scan protocol for whole-cardiac-cycle reconstruction. The exclusion criteria were premature beat, atrial fibrillation, atrial flutter, children and adolescents (age <18 years), and patients with critical illness. Finally, 58 patients were enrolled, of whom 36 underwent CMR (**Supplementary Fig. 1**). The time intervals between CCTA and CMR were ≤24 h (n = 8), 1–7 days (n = 8), 8–30 days (n = 9), and >1 month (n = 11). The cohort included 26 female patients, with an average age of 60.21 ± 14.14 years. The ethics committee of our hospital approved this study (no. 2025-317), and patient informed consent was waived.

### 2.2 Image Acquisition

A 256-slice CT scanner (Revolution Apex CT, GE Healthcare, Milwaukee, WI, USA) with a retrospective electrocardiogram (ECG)-gated scanning protocol was used for CCTA. The scan parameters included a tube voltage of 100–120 kV, a tube current of 800–1000 mAs, a tube rotation time of 0.28 s, and a detector coverage of 140–160 mm. ECG gating covered the entire cardiac cycle (0%–100%). The contrast agent, ioversol (350 mg/100 mL; Hengrui Pharmaceuticals, Lian Yungang, Jiangsu, China), was administered at a dosage of 1.0–1.5 mL/kg and at a rate of 5.0 mL/s after a sequential injection of 10 mL saline, 8 mL contrast at 3.5 mL/s, and 20 mL saline at 3.5 mL/s. A bolus-tracking technique was employed for imaging, with a threshold CT attenuation of 60 HU at the descending aorta and a delay of 8 s after contrast injection.

### 2.3 Image Reconstruction

The reconstructed slice thickness and interval were set to 0.625 mm. Images were reconstructed for the full cardiac cycle (0%–100%) at 5% intervals. Both the standard reconstruction (STD) and SSF2 protocols used deep learning-based image reconstruction. In the STD protocol, images were reconstructed automatically after acquisition. In the SSF2 protocol, STD images were post-processed using the SSF2 software, which performs fully automated reconstruction without interaction. Cardiac phases and reconstruction intervals were kept consistent across both protocols to ensure comparability. SSF2, which operates in axial scanning mode, captures data across three cardiac phases and integrates corresponding images to characterize the motion dynamics of the central phase (the target phase).

### 2.4 Image Quality Assessment

The qualitative evaluation of image quality focused on the clarity of anatomical structures, including the boundaries of the endocardium, valves, papillary muscle, chordae tendineae, and trabeculae. All images were visually assessed and scored on the following scale: 4 = excellent, no artifact; 3 = good, minimal artifact, no impact on diagnosis; 2 = some artifact in the heart, but still usable for diagnosis; 1 = poor, significant artifact, diagnosis not possible. Two radiologists, each with over 5 years of experience in cardiovascular CT post-processing and reporting, conducted the qualitative evaluation. If discrepancies arose, a senior radiologist with over 10 years of experience resolved the inconsistency.

Quantitative evaluation metrics included signal-to-noise ratio (SNR), contrast-to-noise ratio (CNR), edge rise slope (ERS), and entropy. An image with the most severe artifact in the STD protocol was manually selected, the cardiac phase and slice location were confirmed, and the image with the same cardiac phase and slice location of the SSF2 protocol was selected for analysis. A senior radiologist with over 10 years of experience in cardiovascular CT post-processing and reporting conducted the quantitative analysis.

Two regions of interest (ROIs), each measuring 8–10 mm^2^, were placed on the left ventricular myocardium and background, respectively, to calculate the density ratio between the two ROIs and identify SNR. The third ROI of 8–10 mm^2^ was placed on the left ventricle to calculate the density difference between the ventricle and myocardium. This density difference, along with the background density, was utilized to calculate the ratio and obtain CNR.

**Fig. 1.**
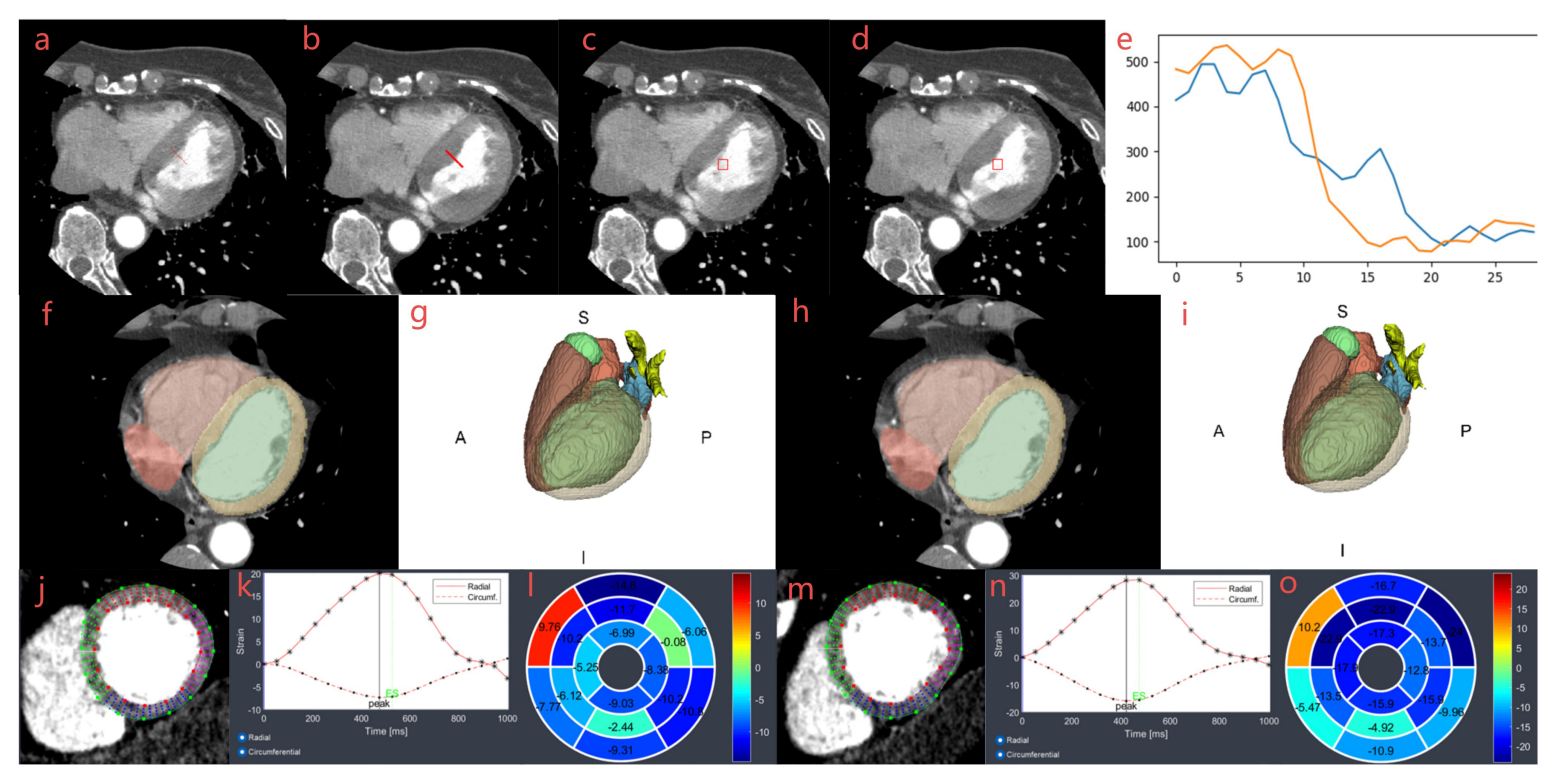
**Workflow of entropy, ERS, heart segmentation, and strain analysis**. Measurement and calculation of entropy (a,b) and ERS (c–e) between STD (a,c) and SSF2 (b,d) cases. ROI and line were drawn manually in (a,c). A matched ROI and line were generated automatically in images (b,d), and two curves in image (e) were used to calculate ERS. Images (f–i) were two-dimensional and three-dimensional segmentations of the heart for STD (f,g) and SSF2 (h,i) cases. Images (j–o) were strain analyses of the left ventricle for STD (j–l) and SSF2 (m–o) cases. Images (j,m) were strain images, images (k,n) were radial and circumferential strain curves, and images (l,o) were strain bullseye maps. A, anterior, S, superior, P, posterior, I, inferior; ERS, edge rise slope; STD, standard reconstruction; SSF2, second-generation snapshot freeze; ROI, region of interest.

A straight line of 2–3 cm length connecting the ventricle and myocardium was drawn to generate a CT attenuation curve at the same location for images reconstructed with the STD and SSF2 protocols (Fig. 1).

A defined ROI of 8–10 mm^2^ was placed on the left ventricle near the endocardium. The entropy can be identified using the following formula:

$$\text{ Entropy }=-\sum_{h\in R\,O\,I}p\,\left(h\right)\,\ln p\,\left(h\right),$$

where *h* is the intensity of a pixel in ROI and p⁢(h) is the probability of the pixels having intensity *h*. The Parzen-window technique was used to estimate p⁢(h) as follows:

$$p\,\left(h\right)=\frac{1}{N}\,\sum_{h_{j}\in R\,O\,I}R\,\left(h-h_{j}\right),$$

where *N* is the number of pixels in the ROI and R⁢(x) is a Gaussian kernel [20].

### 2.5 Automatic Heart Segmentation Using a 3D U-Net Model

In this study, the three-dimensional (3D) U-Net model was employed to automatically segment the heart acquired from STD and SSF2 protocols. The three-dimensional (3D) U-Net architecture comprised three max-pooling and deconvolutional stages, each with a stride of 2 × 2 × 2. The number of convolutional kernels in the contraction path was configured as (16, 32), (32, 64), and (64, 128), with (128, 256) at the bottleneck layer; all used a kernel size of 3 × 3 × 3. The deconvolutional kernels in the expansion path were set to (128, 128), (64, 64), and (32, 11), where “11” corresponds to the number of output volume layers, including background [21]. Based on heart segmentation (Fig. 1), volume-phase curves were generated for the four chambers, and end-diastolic and end-systolic volumes were selected to calculate the ejection fraction.

### 2.6 Manual Segmentation for the Left Ventricle

Left ventricular volume and ejection fraction were analyzed using manual contouring (MC) using the commercially available Segment CT 4.0 (Medviso, Lund, Sweden) software package. Images of the whole cardiac cycle obtained via the SSF2 protocol were subjected to multiplanar reconstruction, and left ventricular short-axis CT cine images were generated with a reconstruction slice thickness and spacing of 3 mm. Endocardial and epicardial borders were manually delineated in the short-axis stack at the end-systolic and end-diastolic phases.

### 2.7 Myocardial Strain Analysis of the Left Ventricle

The commercially available Segment CT 4.0 (Medviso, Lund, Sweden) software package was used to analyze the original three-dimensional CT datasets offline. From these datasets, two-dimensional cine loops of three long-axis views (two-, three-, and four-chamber) and a short-axis stack with a 3-mm reconstruction increment were generated. Strain analysis was performed using reconstructed CCTA cine images. Global longitudinal strain (GLS) and global radial strain for the long axis (GRS-LA) were calculated as the average of peak systolic strain values extracted from three long-axis views (two-, three-, and four-chamber). Global circumferential strain (GCS) and global radial strain of short-axis (GRS-SA) were derived from three short-axis views (basal, mid, and apical; Fig. 1). CVI42 6.0.2 (Circle Cardiovascular Imaging, Calgary, Canada) was used for strain analysis on CMR. End-diastolic contours of the left ventricular endocardium and epicardium were manually delineated on two-, three-, and four-chamber views and short-axis cine images, and myocardial strain was calculated. To ensure reliability, intraobserver and interobserver variability in myocardial strain analysis were assessed before strain analysis for both CCTA and CMR datasets. Observers comprised radiologists with over 10 years of work experience in CCTA and CMR.

### 2.8 Statistical Analysis

Quantitative data were presented as means ± standard deviations, whereas qualitative data were expressed as percentages. The Kolmogorov-Smirnov test was used to assess the normality of data distribution. A paired *t*-test was used to compare the data between two groups. The difference between the image quality scores of the observers was analyzed using the kappa coefficient. Intraobserver and interobserver differences were assessed using the intraclass correlation coefficient (ICC). The correlation of parameters measured using different acquisition protocols was evaluated using ICC, whereas the difference of parameters was assessed with the coefficient of variation (CV). The coefficient of variation is the ratio of the standard deviation to the mean. CV <5%, the agreement is excellent; 5% < CV < 10%, the agreement is good; 10% < CV < 20%, the agreement is clinically acceptable; CV >20%, the agreement is poor. SPSS 20.0 (IBM Corp., Armonk, NY, USA) and MedCalc 20.019 (MedCalc Software Ltd., Ostend, Belgium) were used for statistical analyses and graphs. *p*-values of < 0.05 indicated statistical significance.

## 3. Results

This study enrolled 58 patients with high heart rates, with an average heart rate of 89.40 ± 7.25 beats/min (range: 80–108 beats/min). The average dose length product was 452.06 ± 41.87 mGy × cm). **Supplementary Table 1** presents detailed baseline characteristics.

**Fig. 2.**
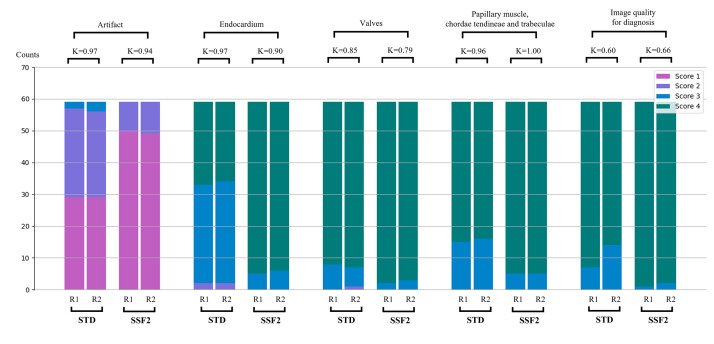
**Image quality and artifact assessment between the STD and SSF2 groups by two readers**. *p* = 0.03 for the comparison between groups, the remaining *p*-values were <0.01 between groups. R1, reader 1; R2, reader 2; 4 = excellent, no artifact; 3 = good, minimal artifact, no impact on diagnosis; 2 = some artifact in the heart, but still usable for diagnosis; 1 = poor, significant artifact, diagnosis not possible. K, kappa value.

**Fig. 3.**
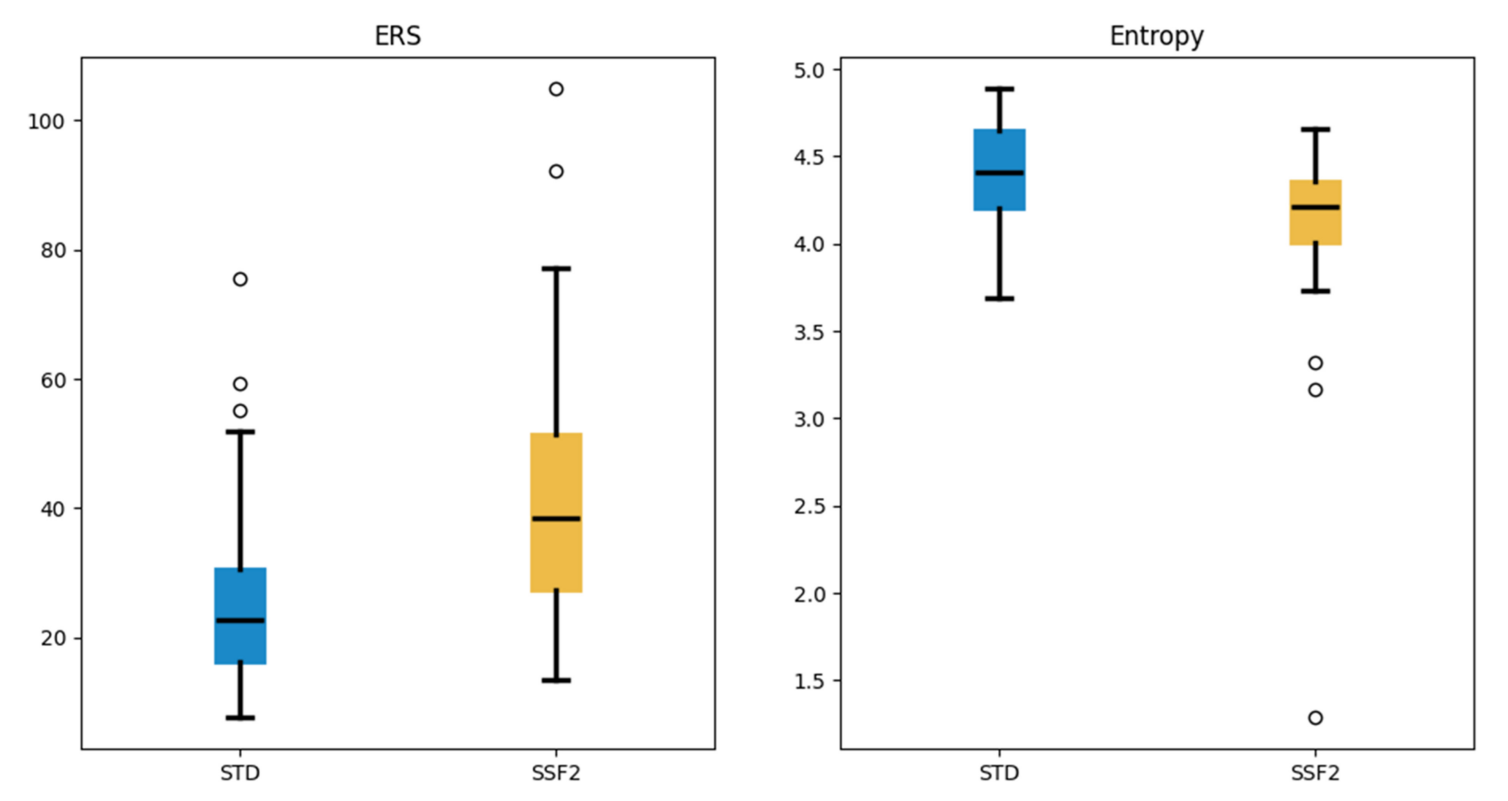
**Comparisons of ERS and entropy between the STD and SSF2 groups**. All *p*-values were <0.01.

The image quality scores for endocardium, valves, papillary muscles, chordae tendineae, and trabeculae were higher; the overall image quality for diagnosis score was higher in SSF2 protocol. (3.91 ± 0.29 vs. 3.84 ± 0.37, *p* < 0.01; Table 1 and Fig. 2). In terms of quantitative parameters, SNR (9.02 ± 4.54 vs. 7.30 ± 4.00, *p* < 0.01), CNR (6.55 ± 3.62 vs. 5.03 ± 3.34, *p* < 0.01), and ERS (41.71 ± 19.03 vs. 25.59 ± 13.16, *p* < 0.01) were higher, whereas entropy (4.12 ± 0.48 vs. 4.40 ± 0.28, *p* < 0.01) was lower in the SSF2 group (Fig. 3).

**Table 1.** **Parameters and scores visual assessment between STD and SSF2 protocols**.

		STD protocol	SSF2 protocol	*p* value
Parameter				
	CT Blood	402.02 ± 92.71	448.79 ± 96.79	<0.01
	CT Myocardium	130.30 ± 55.74	121.30 ± 37.32	0.12
	Background	69.89 ± 34.21	61.70 ± 29.74	<0.01
	SNR	7.30 ± 4.00	9.02 ± 4.54	<0.01
	CNR	5.03 ± 3.34	6.55 ± 3.62	<0.01
	ERS	25.59 ± 13.16	41.71 ± 19.03	<0.01
	Entropy	4.40 ± 0.28	4.12 ± 0.48	<0.01
Visual assessment				
	Artifact	1.53 ± 0.68	1.15 ± 0.38	<0.01
	Endocardium	3.68 ± 0.57	3.93 ± 0.26	<0.01
	Valves	3.86 ± 0.40	3.95 ± 0.23	0.03
	Papillary muscle, chordae tendineae and trabeculae	3.86 ± 0.35	3.96 ± 0.19	<0.01
	Image quality for diagnosis	3.84 ± 0.37	3.91 ± 0.29	<0.01

SNR, signal-to-noise ratio; CNR, contrast-to-noise ratio.

**Fig. 4.**
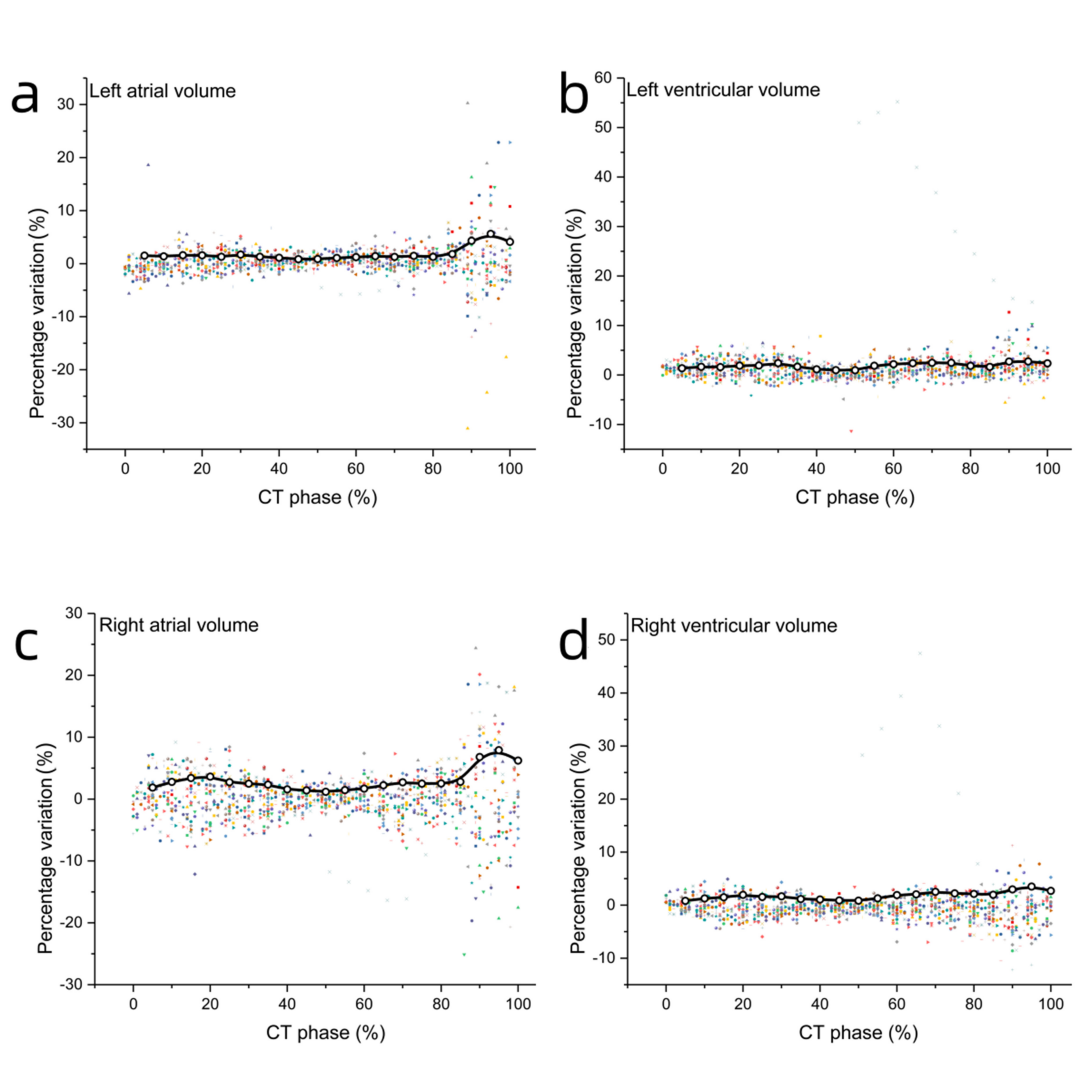
**Percentage variation of left atrial, left ventricular, right atrial, and right ventricular volumes between STD and SSF2 groups**. Percentage variation of chamber volume between STD and SSF2 groups for left atrial volume (a), left ventricular volume (b), right atrial volume (c), right ventricular volume (d), respectively. Percentage variation = (Volume_SSF2_ – Volume_STD_) / Volume_SSF2_.

Automatic segmentation of the left ventricle indicated that left ventricular end-diastolic volume (LVEDV) and left ventricular end-systolic volume (LVESV) were increased in the STD and SSF2 protocols compared with the MC protocol. The ICCs for LVEDV and LVESV were excellent (>0.95), and the CVs of LVEDV was smaller (<9%) (Table 2). The automatic segmentation of the whole heart for the STD and SSF2 protocols indicated that differences of all parameters were small, among which the mean differences in volume were <2 mL, and the mean differences of ejection fraction values were <3% between protocols (Table 3). The volume differences at 90%, 95%, and 100% phases were larger, and the average differences of left ventricular, right ventricular, left atrial, and right atrial volume variation were 2.61% (2.37%–2.75%), 3.06% (2.69%–3.51%), 4.68% (4.12%–5.62%), and 6.98% (6.23%–7.90%), respectively. However, the average differences for 0%–85% phases were 1.80% (0.97%–2.41%), 1.57% (0.82%–2.21%), 1.34% (0.84%–1.81%), and 2.31% (1.18%–3.63%), respectively. The difference in atrial volume was slightly larger than that in ventricular volume (Fig. 4).

**Table 2.** **Comparison of left ventricular volume and ejection fraction between automatic segmentation in STD, SSF2 protocol and MC protocol**.

Variable	STD protocol (n = 58)	SSF2 protocol (n = 58)	MC protocol (n = 58)	STD protocol versus MC protocol			SSF2 protocol versus MC protocol		
				*p* value*	ICC*	CV* (%)	*p* value†	ICC†	CV† (%)
LVEDV (mL)	139.97 ± 43.30	138.30 ± 42.48	132.48 ± 41.72	<0.01	0.96	8.42	<0.01	0.96	7.84
LVESV (mL)	70.25 ± 37.86	70.20 ± 37.99	50.62 ± 37.61	<0.01	0.95	48.24	<0.01	0.95	48.18
LVEF (%)	51.28 ± 7.09	48.86 ± 7.22	64.61 ± 12.33	<0.01	0.59	20.14	<0.01	0.59	21.09

MC, manual contouring; ICC, intraclass correlation coefficient; CV, coefficient of variance. *, STD protocol versus MC protocol; †, SSF2 protocol versus MC protocol.

**Table 3.** **Comparison of atrial, ventricular volume and ejection fraction between STD and SSF2 protocols**.

Variable	STD (n = 58)	SSF2 (n = 58)	*p* value	ICC	95% CI	CV (%)	95% CI
LVEDV (mL)	139.97 ± 43.30	138.30 ± 42.48	<0.01	0.99	0.99–0.99	1.24	1.01–1.48
LVESV (mL)	70.25 ± 37.86	70.204 ± 37.99	0.88	0.99	0.99–0.99	0.78	0.64–0.93
LVEF (%)	51.28 ± 7.09	48.86 ± 7.22	<0.01	0.99	0.99–0.99	1.58	1.29–1.88
RVEDV (mL)	144.75 ± 42.56	145.84 ± 41.83	<0.01	0.99	0.99–0.99	1.79	1.46–2.13
RVESV (mL)	82.75 ± 33.70	83.03 ± 33.96	<0.01	0.99	0.99–0.99	0.64	0.52–0.75
RVEF (%)	43.73 ± 6.84	44.07 ± 6.90	0.06	0.98	0.97–0.99	2.16	1.76–2.57
LAEDV (mL)	90.42 ± 30.57	89.72 ± 30.46	<0.01	0.99	0.99–0.99	0.76	0.61–0.90
LAESV (mL)	47.13 ± 28.62	47.35 ± 28.76	0.17	0.99	0.99–0.99	2.57	2.09–3.06
LAEF (%)	50.47 ± 9.94	49.90 ± 9.78	<0.01	0.99	0.98–0.99	2.35	1.91–2.80
RAEDV (mL)	91.20 ± 32.04	90.58 ± 31.86	<0.01	0.99	0.99–0.99	1.03	0.84–1.22
RAESV (mL)	50.60 ± 22.21	50.63 ± 22.91	0.91	0.99	0.99–0.99	3.54	2.87–4.21
RAEF (%)	45.00 ± 7.42	44.77 ± 7.37	0.52	0.93	0.89–0.96	4.63	3.76–5.52

CV, coefficient of variance; CI, confidence interval; LVEDV, left ventricular end diastolic volume; LVESV, left ventricular end systolic volume; LVEF, left ventricular ejection fraction; RVEDV, right ventricular end diastolic volume; RVESV, right ventricular end systolic volume; RVEF, right ventricular ejection fraction; LAEDV, left atrial end diastolic volume; LAESV, left atrial end systolic volume; LAEF, left atrial ejection fraction; RAEDV, right atrial end diastolic volume; RAESV, right atrial end systolic volume; RAEF, right atrial ejection fraction.

**Fig. 5.**
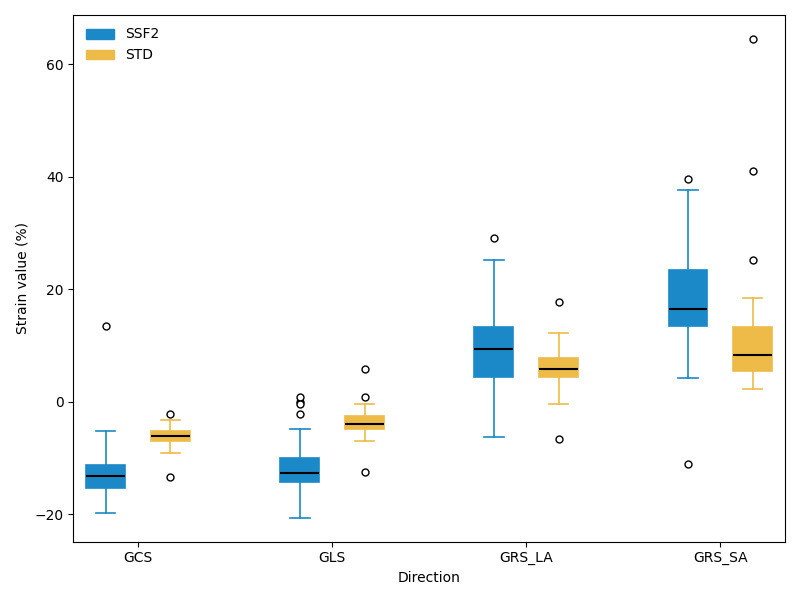
**Comparisons of strains between the STD and SSF2 groups**. All *p*-values were <0.01.

The intraobserver and interobserver difference assessment revealed good myocardial strain reproducibility of Medviso Segment CT for CCTA and Circle CVI42 for CMR, and the ICCs were >0.85. Compared with the STD protocol, the SSF2 protocol demonstrated higher myocardial strain values (–13.26 ± 3.25 vs. –6.01 ± 1.69, *p* < 0.01, 18.33 ± 8.11 vs. 10.60 ± 9.54, *p* < 0.01, –11.89 ± 4.47 vs. –3.92 ± 1.89, *p* < 0.01, and 9.89 ± 6.65 vs. 6.35 ± 3.23, *p* < 0.01 for GCS, GRS-SA, GLS, and GRS-LA, respectively; Fig. 5 and **Supplementary Table 2**). The ICCs of LVEDV and LVESV among the STD, SSF2, and CMR groups were excellent. Compared with the STD protocol, the SSF2 protocol exhibited a better ICC and a smaller CV of myocardial strain with the CMR protocol. Compared with the CMR protocol, the STD and SSF2 protocols demonstrated lower GRS-SA (9.33 ± 6.85 vs. 17.93 ± 7.76 vs. 21.49 ± 9.52) and GRS-LA (6.37 ± 3.62 vs. 10.41 ± 6.66 vs. 17.01 ± 10.44) and higher GCS (–5.99 ± 1.38 vs. –13.30 ± 3.42 vs. –15.01 ± 4.44) and GLS (–4.08 ± 1.99 vs. –11.80 ± 4.83 vs. –13.01 ± 4.36). Considering differences between the STD and CMR protocols, the SSF2 and CMR protocols reached statistical significance (Table 4). The ICCs of GCS, GRS-SA, GLS, and GRS-LA were good between the SSF2 and CMR groups, and the SSF2 protocol demonstrated a smaller CV of myocardial strain with the CMR protocol.

**Table 4.** **Comparison of left ventricular volume, ejection fraction and strain between STD, SSF2 and CMR protocols**.

Variable	STD protocol (n = 36)	SSF2 protocol (n = 36)	CMR protocol (n = 36)	STD protocol versus CMR protocol			SSF2 protocol versus CMR protocol		
				*p* value*	ICC*	CV* (%)	*p* value †	ICC†	CV† (%)
LVEDV (mL)	140.26 ± 46.94	138.29 ± 46.16	130.21 ± 48.89	<0.01	0.97	13.04	<0.01	0.97	12.30
LVESV (mL)	70.17 ± 42.80	70.27 ± 43.06	51.17 ± 42.92	<0.01	0.98	58.98	<0.01	0.98	59.07
LVEF (%)	51.72 ± 7.95	50.99 ± 8.16	64.29 ± 13.74	<0.01	0.70	20.45	<0.01	0.69	21.71
GCS (%)	–5.99 ± 1.38	–13.30 ± 3.42	–15.01 ± 4.44	<0.01	0.30	96.48	<0.01	0.90	14.76
GRS-SA (%)	9.33 ± 6.85	17.93 ± 7.76	21.49 ± 9.52	<0.01	0.44	114.97	<0.01	0.79	29.03
GLS (%)	–4.08 ± 1.99	–11.80 ± 4.83	–13.01 ± 4.36	<0.01	0.31	174.20	0.03	0.85	79.23
GRS-LA (%)	6.37 ± 3.62	10.41 ± 6.66	17.01 ± 10.44	<0.01	0.24	204.27	<0.01	0.65	113.85

GCS, global circumferential strain; GRS-SA, global radial strain of short-axis cine; GLS, global longitudinal strain; GRS-LA, global radial strain of long-axis cine. *, STD protocol versus CMR protocol; †, SSF2 protocol versus CMR protocol.

## 4. Discussion

Accurate cardiac function analysis depends on the reconstruction of the whole cardiac cycle. Incorporating additional reconstruction phases that cover the full cardiac cycle improves functional evaluation accuracy [22]. In addition, high-quality CCTA imaging is crucial for assessing subendocardial diseases, excessive trabeculation, mural thrombus, cardiomyopathy, cardiac tumors, and other conditions. Consensus documents also confirm that CCTA reliably evaluates the quantification of left and right ventricular volumes and ejection fraction, showing excellent agreement with the reference standard CMR [23]. The findings of this study demonstrate that the application of SSF2 significantly enhances image quality in patients with high heart rates and improves CNR between the myocardium and ventricle. This enhancement facilitates improved detection and differentiation of thrombus, as well as accurate assessment of volume and ejection fraction. Moreover, images obtained using SSF2 offer greater reliability for myocardial strain evaluation compared to STD protocol. These results underscore the clinical relevance of SSF2 in the assessment of cardiac structure and function among patients with high heart rate.

When the heart rate is ≥80 beats/min, a prolonged ejection period and shortened filling period would lead to pronounced artifacts. SSF2 has improved valve image quality in patients with high heart rates [3]. However, limited research has addressed the image quality of the endocardium and papillary muscle. High heart rate has been reported to cause endocardium blurring, inaccurate segmentation, and impaired assessment of ventricular volume and function without heart motion correction algorithm [22]. This study is the first to assess the feasibility of SSF2 for full cardiac cycle reconstruction in patients with high heart rates. Our results demonstrate that the SSF2 protocol effectively reduced motion artifacts and improved SNR and CNR of the myocardium and blood pool, resulting in a more uniform and less heterogeneous blood pool. However, in some cases, heterogeneous mixing of contrast medium and blood in the right atrium was observed, which is considered an artifact and incorrectly calibrated with SSF2. To improve the mixing of contrast medium and blood and ensure accurate assessment of cardiac structure and function, dual-bolus contrast media injection or postpositive dual-flow CCTA [24] is recommended.

Manual CCTA segmentation has demonstrated comparable efficacy to CMR in assessing left ventricular ejection fraction [10]. In our study, the difference between automatic segmentation using the SSF2 protocol and manual segmentation is smaller than that observed with the STD protocol. However, the difference in LVESV between the SSF2 and STD protocols is not statistically significant. Notably, the difference in LVESV between automatic and manual segmentation using the SSF2 protocol is statistically significant. Several factors may be associated with this. First, CCTA images predominantly comprise diastolic frames, which are used for training using the 3D U-Net algorithm. Its performance on systolic frames likely suffers due to the unbalanced training dataset. Furthermore, at end-systole, the gap between the papillary muscles, trabeculations, and myocardium is smaller or even absent. Another contributing factor may be the overtracing of the endocardial boundary and misdefinition of the mitral valve annulus plane when using the automatic approach [8].

Compared with automatic segmentation using the STD protocol, the end-diastolic volumes of left ventricle, left atrium and right atrium are larger when using the SSF2 protocol. This difference is primarily due to a clearer and sharper boundary between the endocardium and the blood pool in the SSF2 protocol. Significant differences in automatic segmentation between the SSF2 and STD protocols are observed in the 90%, 95%, and 100% phases, corresponding to the rapid ejection phases of the cardiac cycle. During these phases, the rapid contraction of the ventricles results in more artifacts, highlighting the superior motion correction performance of the SSF2 protocol in these phases.

CT strain analysis is highly variable due to various CT techniques, protocols, and post-processing algorithms [25]. Our results indicate that the strain measurements obtained using the STD and SSF2 protocols are underestimated compared with those derived from CMR. Several factors contribute to these discrepancies. First, myocardial strain analysis depends on precise endocardial tracing, and poor-quality endocardial images and suboptimal automatic segmentation during systole significantly affect results [26]. Second, the reconstructed cardiac phases and time resolution affect strain measurements. CCTA has underestimated GLS compared with speckle-tracking echocardiography [9]. Variations in the reconstruction increments of the R-R interval (5% and 10%) are a significant source of differences in LV and LA CT-FT strain values [22]. The cardiac phases in CCTA images are 21, fewer than the 30 phases in CMR. Third, differences in myocardial strain may originate from variations in imaging modalities and real-time heart rate [22, 26]. The strain results can differ depending on the post-processing software used [16]. Compared with the STD protocol, the SSF2 protocol demonstrated improved ICCs and relatively smaller CVs in comparison with CMR for myocardial strain assessment. However, the CVs for certain strain parameters, particularly GLS and GRS, remained relatively high, indicating notable variability at the individual level. These findings suggest that SSF2-derived strain measurements show good correlation and consistent trends with CMR, rather than strict interchangeability between the two modalities.

### Limitations

This study has some limitations. First, the study was conducted at a single center with a small sample size, and the time interval between CMR and CCTA varies considerably. Second, we excluded control cases with heart rate of <80 beats/min or >120 beats/min, thereby limiting the evaluation of these heart rate ranges. Because CT images in patients with HR <80 are usually excellent in routine clinical practice, and the CT protocol is usually prospective ECG gating to reduce radiation exposure, and cannot reconstructed whole heart phases images. Furthermore, for patients with heart rate >120 beats/min, the currently available cardiac MRI and CT imaging data remain relatively scarce. Third, the modified contrast agent injection protocol employed in this study may contribute to artifacts from the heterogeneous mixing of contrast agent and blood. Fourth, selecting the “worst-artifact” STD image per case inflates the SSF2 benefit for image quality assessment, and a whole-cycle image quality summary for comparison could be fairer. Fifth, manual segmentation and strain analysis are conducted only for the left ventricle because the right ventricle, right atrium, and left atrium are irregularly shaped and less important than the left ventricular assessment. Finally, the minimum interval for automatic full cardiac cycle reconstruction in this study is 5%. Manual reconstruction with smaller phase intervals to increase the number of phases is feasible; however, it is highly labor-intensive and time-consuming.

## 5. Conclusions

The SSF2 protocol significantly improves the image quality for whole-cardiac-cycle reconstruction and cardiac structure and function evaluation in patients with high heart rates. The results of automatic segmentation indicate strong agreement with manual segmentation for LVEDV evaluation, demonstrating considerable potential for clinical evaluation, despite poor agreement for LVESV. The SSF2 protocol outperforms the STD protocol in myocardial strain evaluation, particularly for GCS and GLS, and demonstrates improved correlation with CMR measurements. However, given the relatively high variability observed for certain strain parameters, SSF2-derived strain values should be interpreted with caution and are currently more suitable for comparative or trend-based analysis rather than direct interchangeability with CMR.

## Availability of Data and Materials

The datasets used and analyzed during the current study are available from the corresponding author on reasonable request.
