# Supplementary material for: Cardiac Structural and Functional Evaluation Using a Heart Motion Correction Algorithm for Coronary Computed Tomography Angiography in Patients With High Heart Rates
Source: Rev Cardiovasc Med. 2026 May 18;27(5):48026. doi: 10.31083/RCM48026 (PMC13227394; doi:10.31083/RCM48026)
Supplement: Supplementary file 1 [file 2153-8174-27-5-48026-s1.zip › Supplementary Tables.docx]

Supplementary Table 1 Baseline characteristics of patients with high heart rate.

| Characteristics | Data |
| --- | --- |
| Age (years) | 60.21±14.14 |
| Gender (female) | 26(44.83) |
| BMI (kg/m^2^) | 24.68±3.39 |
| Heart rate (beats/min) | 89.40±7.25 |
| 80-90 beats/min | 33(56.90) |
| 90-100 beats/min | 20(34.48) |
| 100-110 beats/min | 5(8.62) |
| NYHA class |  |
| NYHA=1 | 20(34.48) |
| NYHA=2 | 26(44.83) |
| NYHA=3 | 10(17.24) |
| NYHA=4 | 2(3.45) |
| LVEF using Echocardiography |  |
| LVEF>50% | 56(96.55) |
| LVEF<50% | 2(3.45) |
| Coronary heart disease | 25(43.10) |
| Arrythmia | 7(12.07) |
| Hypertension | 28(48.28) |
| Cardiomyopathy | 2(3.45) |
| Diabetes mellitus | 10(17.24) |

Data are presented as mean ± standard deviation or n (%), BMI, body mass index, NYHA, New York Heart Association, LVEF, left ventricular ejection fraction.

Supplementary Table 2 Comparison of left ventricular strain between STD and SSF2 protocols.

| Variable | STD  (n=58) | SSF2  (n=58) | P value | ICC | 95%CI | CV (%) | 95%CI |
| --- | --- | --- | --- | --- | --- | --- | --- |
| GCS (%) | -6.01±1.69 | -13.26±3.25 | <0.01 | 0.30 | 0.24-0.51 | 81.63 | 62.55-102.94 |
| GRS-SA (%) | 10.60±9.54 | 18.33±8.11 | <0.01 | 0.11 | -0.15-0.36 | 213.30 | 153.37-287.41 |
| GLS (%) | -3.92±1.89 | -11.89±4.47 | <0.01 | 0.05 | -0.21-0.30 | 92.60 | 70.50-117.56 |
| GRS-LA (%) | 6.35±3.23 | 9.89±6.65 | <0.01 | 0.03 | -0.22-0.29 | 177.87 | 129.78-236.02 |

STD, standard, SSF2, second-generation snapshot freeze, ICC, intraclass correlation coefficient, CV, coefficient of variance, CI, confidence interval, GCS, global circumferential strain, GRS-SA, global radial strain of short-axis cine, GLS, global longitudinal strain, GRS-LA, global radial strain of long-axis cine.
