# Supplementary figures and images for: Cardiac Structural and Functional Evaluation Using a Heart Motion Correction Algorithm for Coronary Computed Tomography Angiography in Patients With High Heart Rates
Source: Rev Cardiovasc Med. 2026 May 18;27(5):48026. doi: 10.31083/RCM48026 (PMC13227394; doi:10.31083/RCM48026)

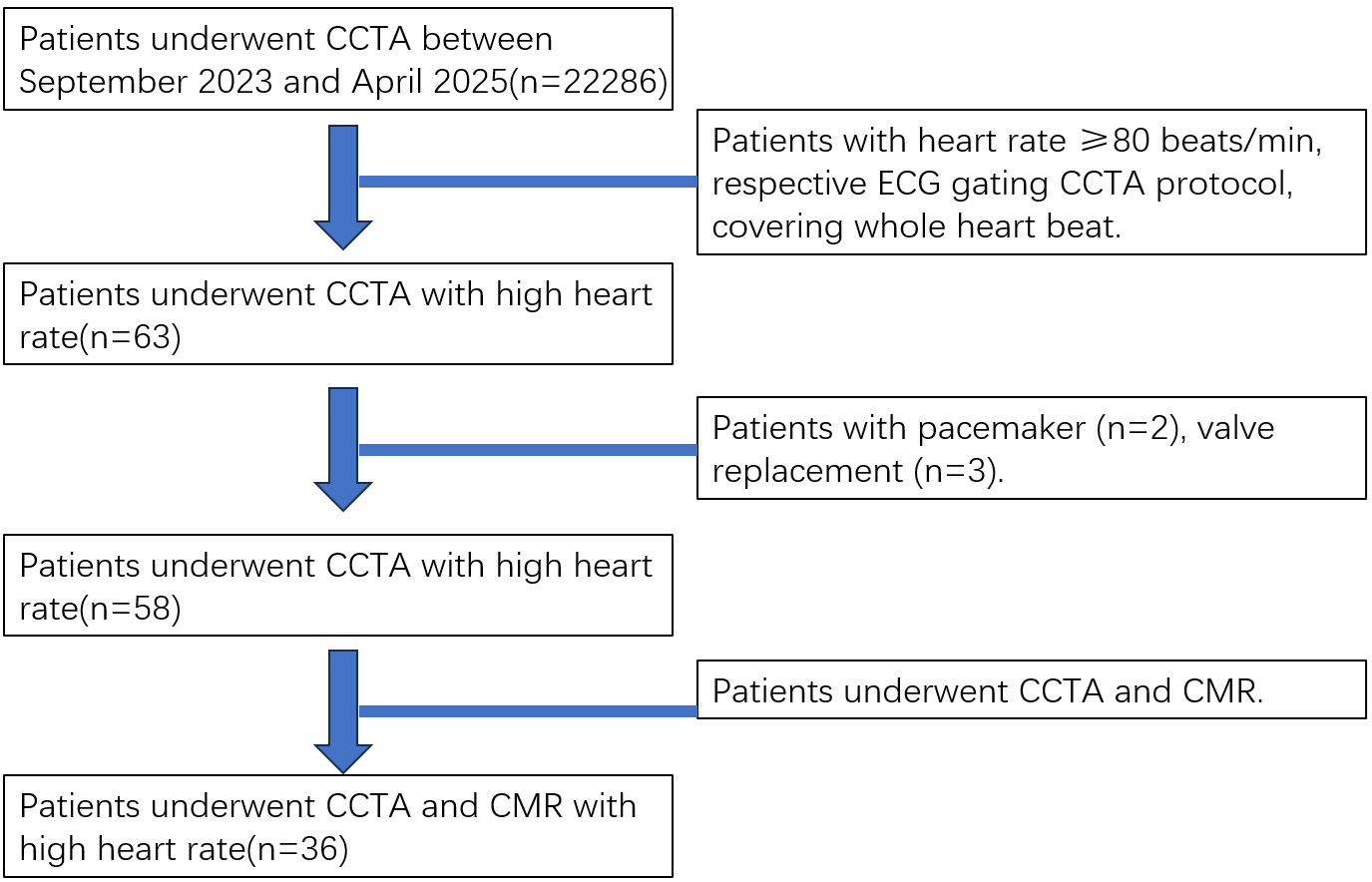

Supplement: Supplementary file 1 [file 2153-8174-27-5-48026-s1.zip › Supplementary Fig. 1.tif]
